# Supplementary material for: Boundary updating as a source of history effect on decision uncertainty
Source: iScience. 2023 Oct 28;26(11):108314. doi: 10.1016/j.isci.2023.108314 (PMC10665832; doi:10.1016/j.isci.2023.108314)
Supplement: Document S1. Figures S1–S3 and Table S1 [file mmc1.pdf]

iScience, Volume *26*

Supplemental information

Boundary updating as a source of history effect on decision uncertainty

Heeseung Lee and Sang-Hun Lee

SUPPLEMENTAL INFORMATION

i

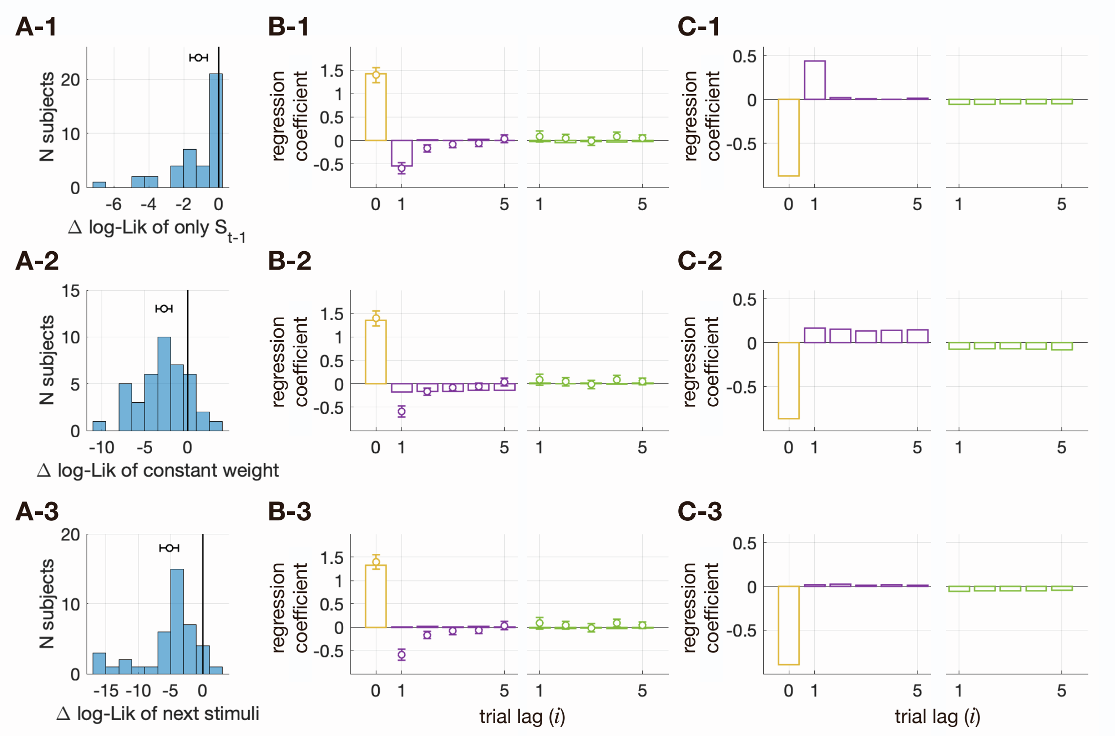

Figure S1. Necessity of the ‘memory-decay’ assumption in explaining the pre-congruence effect, related to Figure 3

To evaluate the necessity of the assumption that the memory recall of a stimulus becomes increasingly noisier as trials elapse (Equations 1 and 2) in accounting for the pre-congruence effect, we created three variants of the original model (BMBU) by modifying the process of boundary inference, as follows. In the first model variant called ‘limited memory access,’ the memory recall was assumed to be accessible only for the immediately preceding stimulus ($\beta_i = 0$ for $i > 1$ in Equation 2). In the second model variant called ‘no memory decay,’ the noisiness of memory recall was assumed not to increase after one trial lag ($\sigma_{r_{t-i}} = \sigma_m(1+\kappa)$ in Equation 1). In the third model variant called ‘prospective memory recall,’ the boundary inference was based on the future stimuli instead of the past stimuli (r_{t-i} is replaced with r_{t+i} in Equation 2). These three model variants were compared with BMBU for goodness-of-fit evaluation, the results of which are summarized in A. The multiple regression analyses depicted in Figure 2C and 3H were repeated using the three model variants, the results of which are summarized in B and C, respectively. The summarized results of the ‘limited memory access,’ ‘no memory decay,’ and ‘prospective memory recall’ model variants are presented in the top (1), middle (2), and bottom (3) rows of panels.

(A) Goodness-of-fit analysis. The histograms show the frequency (number of observers) distributions of the differences in log-likelihood between BMBU and the model variants. For each observer, the predictive power (AIC) of BMBU was subtracted from those of the model variant. The circles and horizontal error bars represent the means and their 95% confidence intervals of the log-likelihood differences. The log-likelihoods of the model variants were all significantly smaller than that of BMBU (‘limited memory access,’ $t = -4.7, (P = 2.7 \times 10^{-5})$; no memory decay,’ $t = -6.2, (P = 2.2 \times 10^{-7})$; ‘prospective memory recall,’ $t = -7.3, (P = 7.9 \times 10^{-9})$).

(B) Model simulation of repulsive bias demonstrated in logistic regressions. The coefficients in the multiple logistic regression of the current choice (C_t) onto the current and previous stimuli (S_{t-i}) and

the previous choice (C_{t-i}) are plotted in circles and bars for humans and the models, respectively. Error bars represent the 95% confidence interval of the means.

(C) Model simulation of pre-congruence and current-congruence effects in linear regressions. The vertical bars represent the coefficients of the multiple linear regression of the simulated decision uncertainty onto the congruences of current choices with the current stimulus ($S_t * C_t$), the previous stimuli ($S_{t-i} * C_t$), and the previous choices ($C_{t-i} * C_t$).

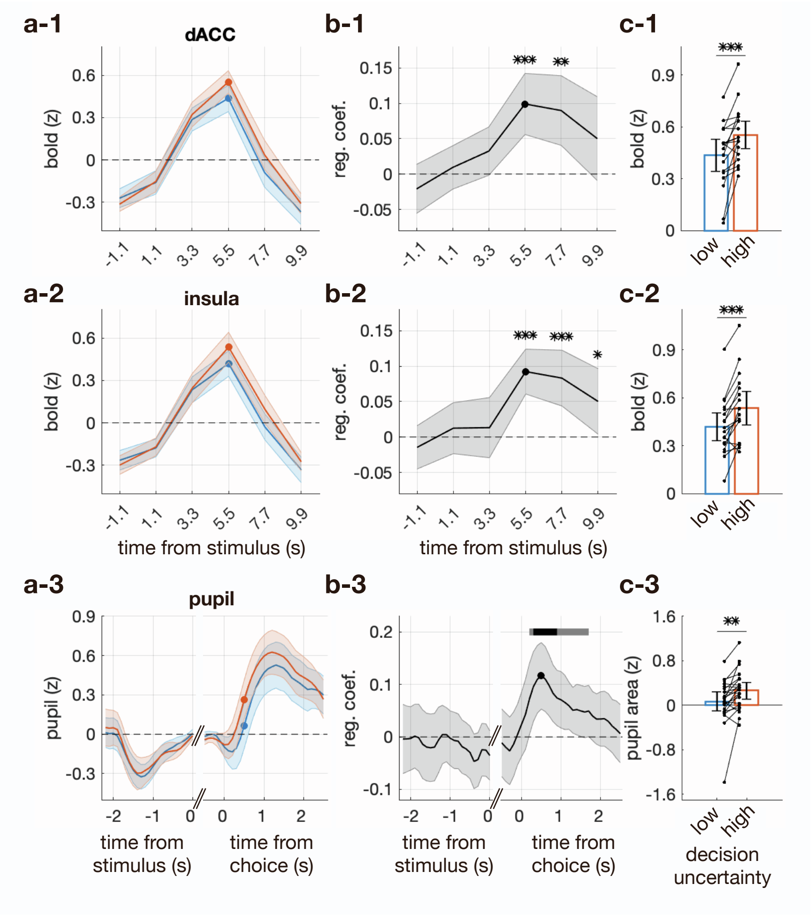

Figure S2. Time courses of the neural and pupillary correlates of the model estimates of decision uncertainty (u_t), related to Figure 7,8

To identify the time points where the neural and pupillary correlates of the model estimates of decision uncertainty (u_t) are maximally pronounced, we carried out the following analyses on the time series of the BOLD responses in the dACC and insula and of the pupil size measurements.

(A) Time courses of BOLD (1,2) and pupil size (3). To visualize the effect of u_t on the BOLD responses and pupil size measurements, we split the trials into the low and high halves in terms of u_t. The colors of the symbols, lines, and shades correspond to the two levels of u_t (blue and red for the low and high levels, respectively). Error bars represent the 95% confidence interval of the means.

(B) Time courses of regression coefficients. We linearly regressed the BOLD responses and pupil size measurements onto u_t in each time point and identified the time points where the significance of those regressions reached their maximum values (the black dots). The coefficients are plotted as a function of time relative to the stimulus onset (1-2) or the time of making choices (3). Error bars represent the 95% confidence interval of the means. The *P* values of two-sided Student's *t*-test of the coefficients are indicated by the horizontal bar (gray: $* < 0.05$; black: $** < 0.01$).

(C) Differences in BOLD (1,2) and pupil size (3) between the two levels of u_t. In the identified time points in B, we compared the signals between trials of low and high halves of u_t. The color scheme was identical to that used in A. Each dot pair with a line represents a single observer. Error bars represent the 95% confidence interval of the means. The asterisks indicate the significance of the difference (*P* values of two-sided Student's *t*-test): $*, P < 0.05$; $**, P < 0.01$; $***, P < 0.001$.

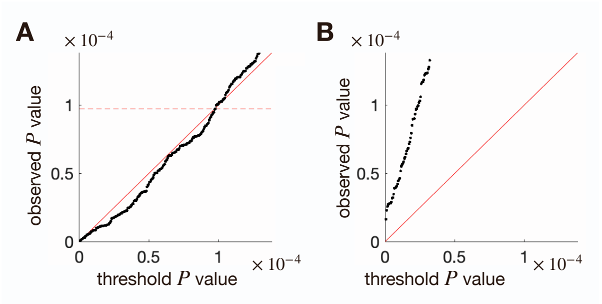

Figure S3. Importance of incorporating the 'boundary updating' process into the definition of decision uncertainty in search of its neural correlate, related to Figure 7

To demonstrate how critical the boundary updating process (as depicted in Figure 3A-E) is for the accurate definition of decision uncertainty, we created a constant-boundary model that lacks this process (see STAR Methods for details) and assessed its effectiveness in discovering BOLD correlates of decision uncertainty. Specifically, we regressed the BOLD responses of all brain voxels onto the model estimates of decision uncertainty as defined by BMBU or the constant-boundary model. We then plotted the lowest *P* values of each voxel against the threshold *P* values to control for the false discovery rate (FDR). By doing so, we could determine the significant voxels after controlling for FDR by defining the critical *P* value, which is the maximum *P* value lower than the FDR threshold (indicated by the horizontal dashed line). When BMBU's estimates of decision uncertainty were used, we found that up to 177 voxels were significant (A), whereas none were significant when the constant-boundary model's estimates of decision uncertainty were used (B).

cortical area	continuous voxel (N)	peak voxel		
		MNI coordinate	t-stat	P-value
dorsal anterior cingulate cortex	64	$[6, 27, 36]$	6.29	8.2×10^{-6}
left insula	27	$[-30, 21, 9]$	6.50	5.7×10^{-6}
right insula	66	$[36, 18, 6]$	7.60	7.5×10^{-7}

Table S1. Brain regions where BOLD responses significantly correlate with the model estimates of decision uncertainty (u_t), related to Figure 7

To define these regions, we used two criteria: (1) the regression coefficient between the BOLD responses and the model estimates of decision uncertainty was significant (two-sided Student's t-test $P < 0.05$, after controlling for the false discovery rate), and (2) there were at least 15 contiguous voxels that met the first criterion.
